# European red squirrel population dynamics driven by squirrelpox at a gray squirrel invasion interface

**DOI:** 10.1002/ece3.1216

**Published:** 2014-09-11

**Authors:** Julian Chantrey, Timothy D Dale, Jonathan M Read, Steve White, Fiona Whitfield, David Jones, Colin J McInnes, Michael Begon

**Affiliations:** 1 Institute of Integrative Biology, University of LiverpoolBiosciences Building, Crown Street, Liverpool, L69 7ZB, U.K; 2 Institute of Infection and Global Health, University of LiverpoolLeahurst Campus, Neston, CH64 7TE, U.K; 3 Lancashire Wildlife Trust, Seaforth Nature ReserveLiverpool, L21 1JD, U.K; 4 Moredun Institute, Pentlands Science ParkBush Loan, Penicuik, Midlothian, EH26 0PZ, U.K

**Keywords:** Ecology, epidemic, epidemiology, infection, mammal, rodent, wildlife

## Abstract

Infectious disease introduced by non-native species is increasingly cited as a facilitator of native population declines, but direct evidence may be lacking due to inadequate population and disease prevalence data surrounding an outbreak. Previous indirect evidence and theoretical models support squirrelpox virus (SQPV) as being potentially involved in the decline of red squirrels (*Sciurus vul*garis) following the introduction of the non-native gray squirrel (*Sciurus carolinensis*) to the United Kingdom. The red squirrel is a major UK conservation concern and understanding its continuing decline is important for any attempt to mitigate the decline. The red squirrel–gray squirrel system is also exemplary of the interplay between infectious disease (apparent competition) and direct competition in driving the replacement of a native by an invasive species. Time series data from Merseyside are presented on squirrel abundance and squirrelpox disease (SQPx) incidence, to determine the effect of the pathogen and the non-native species on the native red squirrel populations. Analysis indicates that SQPx in red squirrels has a significant negative impact on squirrel densities and their population growth rate (PGR). There is little evidence for a direct gray squirrel impact; only gray squirrel presence (but not density) proved to influence red squirrel density, but not red squirrel PGR. The dynamics of red SQPx cases are largely determined by previous red SQPx cases, although previous infection of local gray squirrels also feature, and thus, SQPV-infected gray squirrels are identified as potentially initiating outbreaks of SQPx in red squirrels. Retrospective serology indicates that approximately 8% of red squirrels exposed to SQPV may survive infection during an epidemic. This study further highlights the UK red squirrel – gray squirrel system as a classic example of a native species population decline strongly facilitated by infectious disease introduced by a non-native species. It is therefore paramount that disease prevention and control measures are integral in attempts to conserve red squirrels in the United Kingdom.

## Introduction

Infectious disease is cited as a major factor influencing the population dynamics of coexisting species (Strauss et al. 2012). Apparent competition arises where two species share the same infection (or predator), but differences in pathogenicity (or susceptibility) between hosts may then generate a significant advantage to one species (Prenter et al. 2004). As one host species increases the abundance of the pathogen, this acts to the detriment of the other host species. However, such mechanisms of action have been difficult to demonstrate directly, due to inadequate data on population abundance, infection prevalence, and transmission, especially with respect to wildlife populations (Hudson and Greenman 1998). Thus, where data are limited, there may be an inevitable reliance on mathematical models with estimated parameters and only general empirical support. Current models of the interaction between the gray squirrel (*Sciurus carolinensis*), the red squirrel (*Sciurus vulgaris*), and squirrelpox virus (SQPV), for example, have generally assumed 100% mortality of red squirrels when exposed, and equal infection intensity between the two species, despite subsequent evidence to the contrary (Sainsbury et al. 2008; Atkin et al. 2010; Shuttleworth et al. 2014).

It has been hypothesized that apparent competition through disease is the critical mechanism in the interaction between the invasive American eastern gray squirrel and the native Eurasian red squirrel in the United Kingdom. Their shared pathogen, SQPV, is largely asymptomatic in gray squirrels, yet predominantly lethal in red squirrels (Tompkins et al. 2002). This hypothesis has received strong support from mathematical models. Models only incorporating interspecific competition were unable to explain the rate and pattern of decline of red squirrels in Norfolk (Tompkins et al. 2003) and squirrel distribution data and SQPV infection incidence data matched predictions of infectious disease models in Cumbria (Rushton et al. 2006).

Worldwide, there are few countries with sympatric populations of gray squirrels and red squirrels. The United Kingdom has both species, and since 1944 at least, it has experienced a sustained decline in the native squirrel numbers and distribution, while the introduced gray squirrel population has expanded (Lloyd 1983; Battersby & Tracking Mammals Partnership 2005). Concurrent with this, there have been outbreaks of disease, squirrelpox (SQPx), thought to cause the extinction of local red squirrel populations (Edwards 1962; Sainsbury and Gurnell 1995; Sainsbury and Ward 1996). Regional and national declines in red squirrel numbers have often been attributed to this infectious disease based on anecdotal evidence. Postmortem surveys have reported relatively low levels of confirmed infection (0–18%) in red squirrels (Sainsbury et al. 2008; LaRose et al. 2010; Simpson et al. 2013), although mathematical models show that the impact of an infectious disease on a population can still be critical at low recorded prevalence (Tompkins et al. 2003).

An experimental infection study has shown SQPV to cause severe disease in red squirrels, while no clinical effect is identified in infected gray squirrels (Tompkins et al. 2002). Natural infections in gray squirrels also appear to be predominantly subclinical, with signs of cutaneous lesions being rare (Duff et al. 1996; Atkin et al. 2010). Wild populations of the two species also show dissimilar levels of exposure. In a study of red squirrel cadavers, SQPV antibodies tended to be present only in animals affected by the disease. A few that had no evidence of SQPV infection (8 of 253) showed antibody titers consistent with previous exposure (Sainsbury et al. 2008). This suggests that SQPV infection in red squirrels causes high morbidity and mortality in the wild. SQPV infection in gray squirrels found in the British Isles, however, appears to be endemic, with overall average seroprevalence ranging from 25% to 61%, but locally, prevalence can vary from 0% to 100% (Sainsbury et al. 2000; Bruemmer et al. 2010; Collins et al. 2014).

The effect of SQPV on red squirrel populations has been difficult to demonstrate, in part, due to difficulties in monitoring squirrel abundance directly (Lurz et al. 2008; Gurnell et al. 2009). Previous studies have tended to rely on squirrel presence/absence data or using woodland type to predict squirrel density (Gurnell et al. 2006; Rushton et al. 2006; Sainsbury et al. 2008). The use of such data has failed to identify any link between the infection in the two species of squirrel, with no significant correlation between SQPV infection in red squirrels and either environments capable of supporting both species (Sainsbury et al. 2008) or observed coexistence of the two species (Rushton et al. 2006). More recently, however, the appearance of seropositive gray squirrels and subsequent cases of SQPV infection in red squirrels in Scotland and Ireland (areas that had shown no previous history of SQPV infection) is suggestive of transmission between the two species (McInnes et al. 2009, 2013).

Intensive surveys of natural red squirrel populations (and coexisting gray squirrels) before, during, and after SQPx outbreaks have not previously been carried out. Hence, there is a dearth of data on the associations between SQPx incidence and variations in red and gray squirrel numbers, on patterns of infection during an epidemic, and on survival and recovery within infected red squirrel populations. Here, we present data collected from 2002 to 2012 from before, during, and after an outbreak of SQPx on the Sefton coast, Merseyside, UK. Data on deaths caused by SQPV in this system are combined with line transect survey data to examine associations between SQPV infection and subsequent variations in the abundance of red and gray squirrels. The following predictions are tested: 1) SQPV infection in red squirrels is associated with a reduction in red squirrel density and population growth rate (PGR); 2) gray squirrel density has similar associations; and 3) red squirrel SQPx cases are associated with previous gray squirrel SQPV infection locally. We also investigate whether red squirrels may survive epidemics of SQPx in their natural habitat to play a role in subsequent population recovery.

## Materials and Methods

### Study site

**Figure 1:**
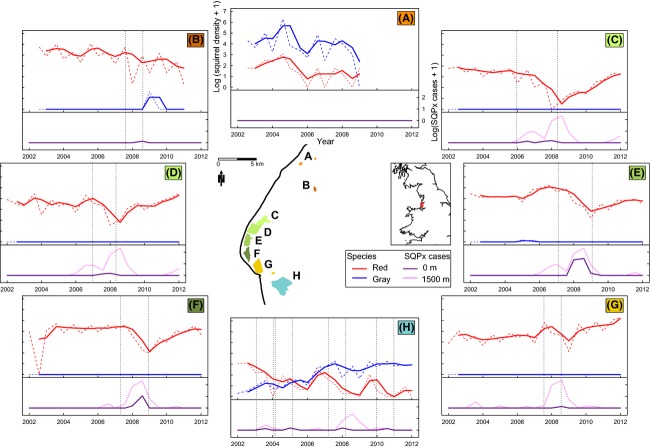
Central map shows area of interest within the United Kingdom and location of study areas; (A) Southport urban (number of transects *t* = 2), (B) Southport rural (*t* = 1), (C) Ainsdale North (*t* = 4), (D) Ainsdale South (*t* = 4), (E) Formby North (*t* = 4), (F) Formby South (*t* = 3), (G) Hightown (*t* = 2), and (H) Ince Blundell (*t* = 5). The surrounding plots show the squirrel densities (mean, weighted by transect length) calculated from transect surveys within that area (upper chart in plot area). The dashed lines indicate actual estimated squirrel densities, while the solid lines show those data with two-point smoothing. Red squirrel pox incidence based on submission of squirrelpox virus (SQPV)-positive carcases is shown in the lower chart in plot areas at 0 m (within study area) and up to 1500 m from the study area (the distance that SQPx cases shown to have the strongest association to red squirrel PGR in subsequent analysis). SQPV-positive cases were assumed to be capable of affecting multiple study areas (no physical barriers were present); therefore, all SQPV-positive cases within 1500 m are plotted for each area irrespective of whether they feature in plots for other areas The vertical gray dashed lines indicate 6 months prior to and after the first and last cases of red squirrel SQPx in that area. Transects are grouped depending on geographic location and whether either or both species were observed.

The Sefton coast, on Merseyside, covers an area of approximately 49 km^2^ (OS Grid Ref: 108:SD275080) (see central map of Fig.1 for study sites). It is a Site of Special Scientific Interest with coastal coniferous woodland in addition to suburban areas, and it is one of the few remaining red squirrel strongholds in England. Since 1993, there has been a gray squirrel control scheme conducted in the urban areas, which in 2005 was expanded to a more systematic control program aimed at forming a refuge for red squirrels surrounded by a buffer zone. This has allowed local red squirrels to live in relative isolation with a much reduced immigrant gray squirrel population.

### Population monitoring

In 2002, the Lancashire Wildlife Trust established 24 line transects in the Sefton Coast area for monitoring squirrel abundance. Five additional transects where added in 2005 (Fig1). Transect length ranged from 600 to 1200 m (mean = 972 m), with an approximate average of 1 m of transect for 400 m^2^ of area represented. Each transect was walked by volunteers three times, twice each year (during March and October), recording the number of squirrels of each species and their perpendicular distance from the transect line. Both transect position and surveyor (where possible) were kept constant throughout the course of the study.

### PostMortem material

From March 2003 to March 2012, red squirrel carcases found by the public or wildlife officers were submitted for routine postmortem examination. Geographic location and date of discovery were recorded. Bodies were either refrigerated at 4°C where necropsy was to be performed within 48 h or stored at −20°C. Tissue samples were taken from all major organs, along with cutaneous samples taken from the face (periorbital and labial) and antebrachium (scent/sensory gland), areas known to harbor large amounts of SQPV in infected animals (Atkin et al. 2010). Routine tissue samples were then frozen at −20 or −80°C and also preserved in 10% formalin. All gray squirrels, culled as part of the control program from March 2003 until October 2009, were also subjected to necropsy examination with blood and major organs being sampled and stored as for the red squirrel tissues.

### SQPV detection

Squirrelpox virus (SQPV) infection of red squirrels was diagnosed initially by the presence of gross lesions (Sainsbury and Gurnell 1995; Sainsbury and Ward 1996). Suspected cases were then confirmed by both identifying histopathological changes consistent with pox viral infection (McInnes et al. 2009) and identifying viral DNA through nested PCR. This used an initial PCR round to identify the target SQPV gene (RNA polymerase subunit RPO147 (Accession Number AY340975). The resulting product was then used as the DNA template for the second PCR round to confirm the correct sequence had been amplified, a product of 275 base pairs indicating a positive result (Atkin et al. 2010). Diagnosis of SQPV infection in gray squirrels relied on the detection of SQPV nucleic acid in any one of the skin samples taken. Nucleic acid was purified from 25 *μ*g of skin using a commercial kit (QIAquick, Qiagen, Sussex, UK) according to the manufacturers' protocol and stored at −20°C.

### Postepidemic survey

Following a SQPx epidemic on the Sefton Coast in 2008, red squirrels were live-trapped throughout the study area, from November 2009 to May 2010, including urban gardens and reserves, covering areas that had previously generated varied numbers of red squirrel SQPx carcases. Squirrels were identified using subcutaneous PIT tags (AVID Plc., Surrey, UK), and blood samples were taken from the greater saphenous vein using aseptic technique. The serum was separated by centrifugation within twelve hours of collection and stored at −20°C. Dacron swabs (Fischer Scientific, Loughbourgh, UK) were used to swab the facial area and antebrachium gland.

Squirrel serum was analyzed for SQPV antibodies using an enzyme-linked immunosorbent assay (ELISA) (Sainsbury et al. 2000). Samples were tested in duplicate with each replicate having its own negative control (reaction well without coated antigen). The average optical density difference (ΔOD) between the antigen-coated and negative controls was measured. A threshold value, indicative of past exposure to SQPV, was identified by taking three standard deviations from the mean of a wholly seronegative population (Greiner and Gardner 2000). Individuals with an ELISA ΔOD over the assigned threshold were also analyzed for the presence of SQPV DNA, as described previously, using the cutaneous swabs and blood cell pellet collected.

### Statistical analysis

Red and gray squirrel density was estimated from population monitoring survey data using the software program Distance 6.0 release 2 (Thomas et al. 2010). This uses the numbers of squirrels observed at different distances from a monitoring transect to fit a detection probability function. This then allowed an estimate of squirrel abundance to be made, using the conventional distance sampling analysis engine. Separate detection probability functions for each transect were generated for each species in each survey period (March or October). However, for an accurate estimation of the probability detection functions, more than 20 observations are required (Buckland et al. 2001). Hence, in order to maintain accurate estimation where observations were less than 20, data were combined across survey periods, and if still required, the observations for both species were combined. The greater accuracy of an estimated probability of detection function that this allowed was seen to outweigh any difference in detection probability of the two species or across the two survey periods. Fifteen transects had sufficient observations to fit separate detection probability functions for the different survey periods and species. Eight of the remaining transects required functions that combined survey periods. Only two required functions also amalgamating both squirrel species. The lowest Akaike information criteria (AIC) were used to select the detection function of best fit. Further details can be found in Buckland et al. (2001) and Thomas et al. (2010).

All other statistical analyses were performed using R version 2.15.1 (R Core Team 2012). To determine the effect of SQPx and gray squirrels on red squirrel abundance and population growth rate (PGR), and the factors leading to SQPx cases, mixed-model linear regression analyses were conducted, with red squirrel density, red squirrel PGR, and red SQPV-infected cases per month as dependent variables. PGRs were calculated from year to year (not survey to survey). For the red squirrel PGR analysis, those data recording repeat zero densities were removed from the analysis, preventing bias created by successive zero data recording a PGR of 0, which would indicate a stable population but was in fact the absence of recolonization in the area. The numbers of SQPx cases associated with each transect was compiled for distances at 500 m intervals from the transect, up to 6 km. Where parameters from previous periods were also included as fixed effects (see below), this was carried out at 6 monthly intervals from 0 to 24 months. With transect as a random effect, model construction was carried out in stages. Each stage began with the optimal model from the preceding stage with the addition of all relevant fixed effects that were subsequently removed or included in a “step-down” manner based on the AIC. The stages were arranged in such a way that maximized data availability and thus model robustness (i.e., missing data increased at each stage). Stage one involved the development of an initial base model investigating the fixed effects of year (to account for annual variation in environmental factors, e.g., weather and food availability), season (April to October and November to March), forestry management activity (past and present), surveyor experience (applicable only with red squirrel density as the dependent variable), and red squirrel population parameters (red squirrel density [present and previous] and red squirrel PGR [present and previous]). Stage two saw the addition of gray squirrel abundance and population dynamics fixed effects (gray squirrel density [present and previous] and gray squirrel PGR [present and previous]). Stage three took the resultant model and investigated red squirrel SQPx cases per month (present and previous) as fixed effects. The final, fourth stage then added gray squirrel SQPV infection status (present and previous) to investigate the role of infection in gray squirrels on red squirrel-dependent variables. In the case of this latter fixed effect, there was potential for confounding variables, as cases of infection in red squirrels may have led to an increase in gray squirrel trapping in the surrounding area. Hence, where numbers of SQPV-positive gray squirrels improved a model, numbers of SQPV-negative gray squirrels were also tested. If this led to a greater or equal reduction in AIC, gray squirrel SQPV infection status was discounted. The number of SQPV-infected carcases was used for both species rather than the prevalence of infection. Carcase recovery rates in the vicinity of the transects were often relatively low, meaning prevalence estimates were often based on a single carcase, giving an inaccurate account of overall prevalence. It is accepted that differences in the number of carcases from differing sites may have been a function of different recovery probabilities. However, this was assumed to vary as a factor of each transect and there using mixed-model regression with transect as a random effect, this variation in carcase recovery rate was taken into account. After each step, when a fixed effect was proved to be significant, then all previously removed fixed effects were tested individually until no improvement to the model could be made resulting in a final optimal model. Error structures were determined prior to analysis as outlined by Bolker et al. (2009); negative binomial with zero inflation, Gaussian, and negative binomial were used for red squirrel density, red squirrel PGR, and red squirrel SQPx cases per month as dependent variables, respectively. A log link function was used, and Laplace approximation applied for model fitting. All regression analyses were performed using the package AD Model Builder (glmmADMB) (Bolker et al. 2009; Fournier et al. 2011).

## Results

### Squirrel densities and PGR

Between March 2003 and March 2012, 1286 line transect surveys were undertaken on 25 transects, each of which was surveyed at least 30 times. Six transects recorded gray and red squirrel presence on more than one survey period, eighteen recorded red squirrel presence only (or gray squirrels only once), and one recorded gray squirrel presence only. Estimated red squirrel densities ranged from 0 to 436 squirrels/km^2^ (mean 42.29; quartiles 0.73, 16.35, and 54.01), and estimated gray squirrel densities from 0 to 541 squirrels/km^2^ (mean 5.80; quartiles 0.00, 0.00 and 0.00). Estimated red squirrel PGR ranged from −0.84 to 0.66 (mean −0.021; quartiles −0.13, 0.00 and 0.10) and gray squirrel PGR from −0.66 to 0.57 (mean −0.0012; quartiles 0.00, 0.00, and 0.00). Overall, the red squirrel population on Merseyside was generally stable until a sudden, large decline of 87% (based on a weighted mean across all study areas) between autumn 2007 and March 2009 (Fig.1).

### SQPx cases

A total of 448 red squirrel carcases were submitted during the course of the study. SQPV infection was diagnosed in 187 individuals, of which 151 (81%) had died directly from the infection and 33 (18%) had died from other causes but were PCR positive for SQPV. Three carcases showed no lesions or overt cause of death but were SQPV PCR positive. Only individuals with a location record accurate to 50 m and a record of the date found were included in the regression analysis (148 of 187). These data are also summarized in Fig.1. There was a large increase in the number of SQPx cases at around the time that the red squirrel population declined. Prior to the main decline (2003–2006), an average of 5.75 SQPx cases per year were submitted, compared with 73.50 cases per year during 2007–2008 and 3.00 cases per year during 2009–2012.

Between March 2003 and October 2009, 309 gray squirrel carcases were submitted, of which 52% (160) underwent PCR analysis for SQPV, and of these, 27% (43 of 160) were positive. Eighty-one negative individuals and 31 positive individuals had spatial and temporal records appropriate (see above) to indicate gray SQPV status in the regression analysis. There was no correlation between the overall SQPV infection prevalence in the two species.

### Regression analysis

Abbreviations used throughout the analyses are summarized in Table1.

**Table 1:** Abbreviations used in GLMM analysis

Parameter	Abbreviation
Year	yr
Season	seas
Red squirrel density *t* months previously	rd_*t*_
Red squirrel population growth rate at *t* months previous	rPGR_*t*_
Gray squirrel density *t* months previously	gd_*t*_
Gray squirrel population growth rate at *t* months previous	gPGR_*t*_
Gray squirrel presence at time *t* months	gpres_*t*_
Red SQPx cases per month at *t* months previously and at *d* meters	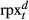
Gray squirrel positive SQPx cases per month at *t* months previously and at *d* meters	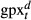

### Red squirrel density

With transect as a random effect, the base model included year and season (October density was greater than in March). In the final model, red squirrel density was associated with gray squirrel presence 24 months previously, gray squirrel PGR 12 months previously, red squirrelpox cases 6 months previously, and contemporary gray squirrelpox cases, all of which had a negative influence (Table2).

**Table 2:** Optimal model of red squirrel density, with parameter coefficients shown. The ΔAIC values displayed are those achieved when the explanatory variable is dropped from the optimal model

Factor	Red squirrel density					
	AIC	ΔAIC	Coefficient	SE	*Z*-value	*P*-value
Intercept	1743.55	–	3.6	0.28	12.75	<2e-16
Yr						
2005	1764.32	20.78	0.17	0.18	0.96	0.34
2006	–	–	0.25	0.17	1.45	0.15
2007	–	–	0.36	0.17	2.12	0.034
2008	–	–	−0.50	0.26	−1.88	0.060
2009	–	–	−0.53	0.22	−2.39	0.017
Seas	1774.77	31.22	−0.62	0.10	−5.98	2.3e-09
gpres_24_	1752.34	8.79	−1.5	0.47	−3.17	0.0015
gPGR_12_	1746.36	2.81	−3.6	1.7	−2.19	0.029
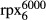	1751.83	8.29	−0.11	0.037	−3.02	0.0026
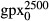	1746.15	2.60	−1.4	0.68	−2.02	0.043
(1|transect)	1879.05	135.51	–	–	–	–

### Red squirrel PGR

With transect as a random effect, the base model included year, previous red squirrel densities (6–24 month previously). In the final model, red squirrel PGR was negatively associated with red squirrel density twelve months previously, red SQPx cases 6 months previously, and the presence of SQPV in gray squirrels, but positively associated with gray squirrel PGR and with red squirrel density 2 years previously (Table3).

**Table 3:** Optimal model of red squirrel PGR, with parameter coefficients shown. The ΔAIC values displayed are those achieved when the explanatory variable is dropped from the optimal model

Factor	Red squirrel PGR					
	AIC	ΔAIC	Coefficient	SE	*Z*-value	*P*-value
Intercept	−403.81	–	0.0022	0.017	0.13	0.90
yr						
2005	−403.71	−0.11	0.011	0.022	0.50	0.62
2006	–	–	0.012	0.021	0.57	0.57
2007	–	–	−0.0022	0.020	−0.11	0.91
2008	–	–	−0.041	0.022	−1.83	0.067
2009	–	–	0.039	0.025	1.59	0.11
rd_12_	−391.62	12.19	−0.00045	0.00012	−3.84	0.00012
rd_24_	−396.56	7.26	0.00034	0.00011	3.08	0.0021
gPGR_0_	−386.56	17.26	0.44	0.098	4.50	6.9e-06
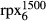	−373.41	30.40	−0.026	0.0044	−5.93	2.9e-09
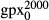	−392.64	11.17	−0.16	0.042	−3.69	0.00022
(1|transect)	−405.81	−2.00	–	–	–	–

### SQPx in red squirrels

With transect as a random effect, the base model included year and red squirrel density 24 months previously. Red squirrel pox cases were positively associated with red SQPx cases 6 and 12 months previously and with gray SQPV-positive squirrels 6 months previously, and negatively associated with red SQPx cases 18 and 24 months previously (Table4).

**Table 4:** Optimal model of red squirrel SQPx cases per month, with parameter coefficients shown. The ΔAIC values displayed are those achieved when the explanatory variable is dropped from the optimal model

	Red squirrelpox cases at 1000 m					
AIC	ΔAIC	Coefficient	SE	*Z*-value	*P*-value
Intercept	137.11	**–**	−22.11	5573	0.00	1.00
yr						
2005	201.80	64.69	17.13	5573	0.00	1.00
2006	–	–	18.50	5573	0.00	1.00
2007	–	–	17.28	5573	0.00	1.00
2008	–	–	19.18	5573	0.00	1.00
2009	–	–	1.81	5573	0.00	1.00
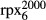	178.90	41.79	0.67	0.13	5.31	1.1e-07
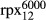	168.49	31.38	1.93	0.34	5.64	1.7e-08
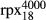	140.50	3.39	−1.26	0.61	−2.07	0.038
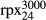	140.72	3.61	−6.05	3.15	−1.92	0.054
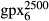	139.51	2.40	1.46	0.78	1.87	0.061
(1|transect)	135.11	−2.00	–	–	–	–

### Postepidemic serology

In total, the sera of 93 live red squirrels were analyzed for SQPV antibodies. Six individuals showed OD values greater than the threshold defining infection. Five of these had *R* values >3.9 at a confidence level of 0.005 using generalized extreme studentized deviate analysis, indicating that they were outliers in comparison with the rest of the data (Rosner 1983). None of the cutaneous swabs or blood cell pellets from these individuals showed evidence of SQPV by PCR.

## Discussion

This research provides strong statistical support for several previously hypothesized but largely untested theories concerning SQPx epidemics in red squirrels, for example that gray squirrels are involved in initiating outbreaks. Red squirrelpox disease transmission has been shown to be dependent on previous historical and local cases and that density-dependent transmission is a likely driving force in maintaining epidemics. These are significant novel findings from this data analysis and the best support yet for our current understanding of squirrelpox epidemiology. The presence of live seropositive wild red squirrel survivors after a pox epidemic is also an entirely new finding.

From 2002, red and gray squirrel density estimates indicate that the red squirrel population on Merseyside was generally stable until a sudden decline of 87% between autumn 2007 and March 2009. This population crash coincided with the appearance of large numbers of red squirrel carcases infected with SQPV, and we provide evidence for an epidemic occurring in the red squirrel population. In the regression analyses, the number of SQPx cases in red squirrels was strongly implicated in variations in red squirrel density and PGR, with an effect delay of around 6 months, thus supporting our first prediction. There were also associations between gray squirrel SQPV-positive cases and red squirrel abundance and PGR, although these were presumably indirect, related to the introduction of SQPV into the red squirrel population. Overall, therefore, our data indicate that the red squirrel population declines were due, to a significant extent, to animals dying from SQPV infection. Thus, through using quantitative survey and mortality data, this study generally supports the findings of previous studies, based on anecdotal and opportunistic survey data, that infectious disease, introduced by the non-native gray squirrel, is a major determinant of red squirrel abundance and distribution (Tompkins et al. 2003; Rushton et al. 2006; Bell et al. 2009; Carroll et al. 2009). However, it also highlights potential inaccuracies in the assumptions made by previous modeling studies, assuming, for example, equal red and gray squirrel infectivity and 100% mortality of SQPV-infected red squirrels.

The role played by direct competition from gray squirrels and the implication for this role more broadly in the United Kingdom are less well defined. This study failed to validate the prediction that gray density significantly impacts red squirrel abundance or population dynamics. Previous empirical studies have cited reduced red squirrel juvenile recruitment and fecundity in the presence of gray squirrels as evidence of competition between the two species (Wauters et al. 2000; Gurnell et al. 2004). Assuming that any effect of direct competition by gray squirrels on red squirrels would be density dependent, there is little evidence to suggest that this played a key role in our system on Merseyside; the only negative effect of gray squirrel population parameters was that of gray squirrel presence 2 years previously and gray squirrel PGR 1 year previously, both on red squirrel density. It should be noted that gray squirrels were absent from many of the areas studied (6 of 8), and due to the predominant coniferous habitat, and the control program, densities were relatively low compared with solely broadleaf woodland or uncontrolled gray squirrel populations (Lurz et al. 1995). Nonetheless, there is evidence (area H, see Fig.1) that red squirrels are capable of population increases in the face of gray squirrel presence when no SQPV infections are recorded in the local red squirrel population; declines only occurred with the appearance of the pathogen in red squirrels.

This is the first account of SQPV infection (as opposed to previous exposure) in gray squirrels being associated with SQPV infection in native red squirrels, thus supporting our final prediction. Indeed, the indicated time lag of 2 months between infection in the two species, and the greater coefficient displayed by the infected gray squirrels compared with the equivalent red squirrel parameter (1.46 [*P *=* *0.061], compared with 0.67 [*P* = 1.1 × 10^−7^] for red squirrel SQPx cases 6 months previously), both support a scenario in which the gray squirrel is responsible for initiating the infection in red squirrels. Prevalence of infection in gray squirrels was generally low, rates of viral shedding from infected gray squirrels are typically very low (Atkin et al. 2010), and the improvement to the regression model from the inclusion of infected gray squirrels, while significant, was relatively slight. By contrast, prevalence of infection in red squirrels during the period of population decline was higher, rates of viral shedding from infected reds are typically very much higher (Atkin et al. 2010), and the improvement to the regression model from the inclusion of infected reds was marked. Thus, once the disease was introduced, the main driver of red squirrel SQPx cases was infection in conspecific red squirrels. A similar conclusion has been drawn from data in Ireland (McInnes et al. 2013). A temporal and spatial trend is also indicated by the regression analysis of red squirrel SQPx cases. This suggests that cases within 12 months up to 6000 m away drive further cases (suggesting a relatively fast transmission rate over the landscape), whereas cases beyond this timescale (18–24 months previously) within a closer vicinity (up to 3000–4000 m away) were negatively associated with subsequent cases. This is consistent with an epidemic pattern with a peak in infection over a 12-month period and then a subsequent trough 18–24 months later, presumably due to high mortality leaving a low density population of red squirrels unable to sustain the pathogen beyond 12 months. This possible density dependence is also indicated by the inclusion of red density 2 years previously in the selected model (before the consideration of the effect of red squirrel SQPx cases). Density-dependent disease transmission among red squirrels, and therefore a threshold abundance (Lloyd-Smith et al. 2005), may give rise to potential opportunities for disease control by reducing the density of susceptible hosts (e.g., through vaccination or habitat management). However, with no good data on actual time at which our red squirrel populations were exposed, no accurate estimation of a threshold density can be made from the current dataset. Nevertheless, based upon these conclusions, the following four suggestions are made for guidance of fieldworkers dealing with red squirrel population management during SQPx outbreaks.

1. An initial assessment should be carried out as to whether any intervention is likely to be effective or ethical. If gray squirrels are prevented from recolonizing the area, then red squirrels may return from surrounding areas, or by surviving exposure to the virus. Thus, the control of the invading (or existing) grays should be perhaps considered the minimum intervention necessary. If not, red squirrels are unlikely to survive in sufficient numbers to recolonize an area. This is especially important where populations are small and fragmented, where low numbers of individuals survive, and where the absence of a surrounding uninfected population makes recolonization unlikely.
2. The success of any intervention will be dictated by the early detection of SQPV in a red squirrel population. The first control measure conducted should be surveillance of both mortality and health of remaining red squirrel populations. This can be by the form of passive carcase collection (as in this study), but more active surveillance is recommended through more systemic surveys, where resources allow. More intensive disease surveillance programs (e.g., live trapping) should be adopted where resources allow, using strict biosecurity protocols.
3. Once SQPV is confirmed in a population, the aim should be to limit the spread of disease as much as possible with the removal of infected red squirrels in the vicinity. Methods on how this is performed will be dependent on the availability of resources and local opinion. Control of disease in wild animals often involves culling of infected individuals and this could be considered, especially as recovery of red squirrels with SQPx is rare in captivity. The evidence of this study would suggest those squirrels that do survive SQPx pose no further risk to naive red squirrels, with no lesions being noted on the surviving individuals and no further pox cases were identified from the areas where the survivors originated.
4. Any practice that has the effect of increasing squirrel densities and increasing the interaction between conspecific red and gray squirrels (e.g., supplementary feeding) should ideally avoided at anytime, but especially during periods of disease outbreak. Evidence suggests that the disease has a direct transmission route and environmental contamination is the main route of transmission.

Modelling studies have tended to neglect the potential for red squirrels to survive SQPV infection, with the exception of one (Gurnell et al. 2006), although in an experimental infection study, the single survivor (of four) displayed lesions for over 6 weeks, during which time it had the potential to transmit SQPV to conspecifics (Tompkins et al. 2002). Furthermore, live captive animals and wild red squirrel carcases have also shown evidence of SQPV exposure, but no concurrent clinical signs (Sainsbury et al. 2008; Shuttleworth et al. 2014). Here, the serological survey adds to previous work and leaves little doubt that red squirrels can survive exposure to SQPV in their natural habitat. Moreover, the data present provide an important opportunity to estimate the approximate proportion of exposed individuals that these survivors represent. From March 2009 (the end of the SQPx epidemic) until the end of the field survey, the red squirrel population in the area of known exposure to the infection (area E, see Fig.1) grew by a factor of 2.6. Thus, the 41 animals captured there in March 2010 can be said to have increased from 16 individuals immediately after the epidemic. During the epidemic (from October 2007 to March 2009), the population at this site suffered a reduction to 6% of its original density. Thus, 16 individuals postepidemic equates to 267 individuals prior to the epidemic, of which 251 died. Of the 16 survivors, five showed evidence of exposure to SQPV, so we assume that the remaining 11 must have survived having previously avoided exposure.

What, although, were the causes of death of the 251 that died? Of red squirrel carcases submitted from within or very close to this area during the period of decline, 82% (72/87) were overtly SQPx positive. Therefore, of 251, 206 deaths (82%) can be attributed to SQPx. However, of the remaining 45 that died of other causes, some will previously have survived exposure to SQPV. The proportion doing so should be the same as the proportion of subsequent survivors that previously survived exposure to SQPx (5 of 16), giving 14 individuals (of 45) that survived exposure, but then died of other causes. Thus, we have a total number exposed of 225 (206 died, 14 survived, but died subsequently, and 5 survived ultimately), an exposure rate of 84% (225 of 267), and a survival rate following exposure of 8.4% (19 of 225) (see table5 below).

**Table 5:**
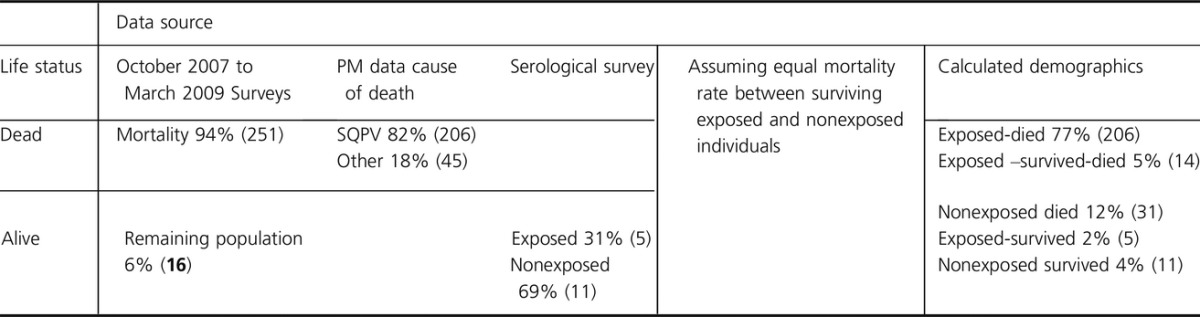
Calculation of the predicted exposure and survival rates of squirrelpox virus (SQPV) at site E. The numbers in brackets are the predicted numbers of individuals from the equivalent sample population derived from 41 individuals caught postepidemic from October 2009 to March 2010. This equates to 16 individuals (number in bold) in March 2009 after the population grew by a factor of 2.6 from March 2009 (end of SQPx epidemic) to March 2010 (when sampling ceased)

Whether this offers hope for the recovery of red squirrel populations following epidemics, perhaps with increasing proportions of resistant individuals, or may result in greater overall mortality as some theory suggests (Gurnell et al. 2006), remains uncertain. Current indications are that the red squirrel population on Merseyside has attained similar levels to those prior to the SQPx epidemic. This suggests that if gray squirrels are prevented from colonizing an area after a SQPx epidemic, red squirrel populations can recover. Ultimately, these red squirrels will form the genetic basis for the descendant population and a degree of resistance should evolve over time resulting from this strong selection pressure. Subsequent outbreaks may further promote this process as long as sufficient survivors remain to re-establish the population.

Our study has confirmed that SQPx can influence red squirrel numbers beyond any direct effect of gray squirrels and that SQPV is a major concern for both the continued presence and welfare of the red squirrels that still remain in the United Kingdom. It is in general agreement with modelling studies that have preceded it (Tompkins et al. 2003; Rushton et al. 2006; Bell et al. 2009) and has highlighted further important features such as the survival of exposed individuals and the relative importance of gray and red squirrels in initiating and sustaining epidemics, respectively. This latter point is important because gray squirrel control programs cannot stop the influx of every migrating gray squirrel. Thus, while this currently remains the only control measure, SQPx epidemics are to be expected in red squirrels, especially where their densities are high. Where outbreaks do occur, in order to mitigate the effect of the pathogen on a red squirrel population, both removal of all gray squirrels and infected red squirrels will be required, but such measures will be dependent on early detection of such an event. Hence, it is of paramount importance that there is adequate disease surveillance of remaining red squirrel populations.

Overall, the study on Merseyside has shown there is potential for the continued survival of red squirrels on the mainland in the United Kingdom. However, this will require resource intensive programs that involve reducing the interaction between red and gray squirrels, which will depend heavily on both local and national (NGO and even governmental) support. The success of such programs will hinge on sound disease control or prevention measures, for which there is still a large requirement for further knowledge on the epidemiology of SQPV and other squirrel diseases. There is now a need to confirm the general conclusions herein with similar studies in other areas of the United Kingdom to differentiate, if present, any local variation in the effect of disease of red squirrel populations in the United Kingdom and potentially Europe.
